# A structural analysis of the hypoxia response network

**DOI:** 10.7717/peerj.10985

**Published:** 2021-04-06

**Authors:** Jianjie Li, Yuqi Gao, Xuan Yu

**Affiliations:** 1 Department of Health Service, Army Medical University, Chongqing, Shapingba, China; 2 Institute of Medicine and Hygienic Equipment for High Altitude Region, Army Medical University, Chongqing, Shapingba, China

**Keywords:** Hypoxia response network, Complex networks, Network topology, Bow-tie structure, Structural complexity

## Abstract

**Background:**

The hypoxia-inducible factor-1 (HIF-1) signaling pathway is an important topic in high-altitude medicine. Network analysis is a novel method for integrating information on different aspects and levels of biological networks. However, this method has not been used in research on the HIF-1 signaling pathway network. To introduce this method into HIF-1-related research fields and verify its feasibility and effectiveness, we used a network analytical method to explore the structural attributes of the HIF-1 signaling pathway network.

**Methods:**

First, we analyzed the overall network of the HIF-1 signaling pathway using information retrieved from the Kyoto Encyclopedia of Genes and Genomes (KEGG). We performed topology analysis, centrality analysis, and subgroup analysis of the network. Then, we analyzed the core network based on the overall network analysis. We analyzed the properties of the topology, the bow-tie structure, and the structural complexity of the core network.

**Results:**

We obtained topological structure diagrams and quantitative indicators of the overall and core networks of the HIF-1 signaling pathway. For the structure diagrams, we generated topology diagrams of the network and the bow-tie structure of the core network. As quantitative indicators, we identified topology, centrality, subgroups, the bow-tie structure, and structural complexity. The topology indicators were the number of nodes, the number of lines, the network diameter, and the network density. The centrality indicators were the degree, closeness, and betweenness. The cohesive subgroup indicator was the components of the network. The bow-tie structure indicators included the core, input, and tendril-like structures. The structural complexity indicators included a power-law fitting model and its scale parameter.

**Conclusions:**

The core network could be extracted based on the subgroup analysis of the overall network of the HIF-1 signaling pathway. The critical elements of the network could be identified in the centrality analysis. The results of the study show the feasibility and effectiveness of the network analytical method used to explore the network properties of the HIF-1 signaling pathway and provide support for further research.

## Background

Hypoxia affects the work abilities and health of soldiers at high altitudes (Truesdell & Wilson, 2006; Qiang et al., 2014). Research on the hypoxia response network (HRN) is a necessary field of high-altitude medicine that aims to promote the health and work abilities of soldiers at high altitudes. The hypoxia-inducible factor-1 (HIF-1) signaling pathway is one of the crucial components of the HRN (Harris, 2002; Hockel & Vaupel, 2001). Most studies on the HRN have been carried out based on classical biomedical methods, and these studies have made remarkable achievements. With the development of systems biology, researchers have tried to explore the HRN from a systematic view to compensate for the shortcomings of traditional research methods. Kohn et al. (2004) proposed a theoretical HRN model based on ordinary differential equation behavior. They analyzed the structural composition of HRN in detail with the aim of identifying the core subsystem responsible for HRN conversion. The network has been decomposed into multiple primary paths using the extreme path analysis method (Palsson, 2006). It can be well matched with the consensus of existing research, showing that the path switching or branching effect may be the cause of the intense response to the oxygen concentration. Heiner & Sriram (2010) subsequently constructed the general Petri net structural model of the HRN and analyzed the relative modules and properties of the network algorithmically. Additionally, network analysis methods and tools have been used to analyze biological networks, providing a novel perspective for studying traditional biological problems (Barabasi & Oltvai, 2004). Ding et al. (2009) studied the structural and functional properties of the giant strong component of the *B. thuringiensis* metabolic network. Zhang & Zhang (2019) performed a protein-protein interaction network analysis of insecticide resistance molecular mechanisms. Ma & Zeng (2003) studied the connectivity of the metabolic networks of 65 biological species, which did not include the core network of HIF-1 signaling pathways. They found that these biological metabolic networks are similar to the Internet in their macrostructure, which also presents a bow-tie structure (Ma & Zeng, 2003). Network analysis methods focus on the properties of the connections of things and investigate these connections as a whole (Boccaletti et al., 2006). However, this method has not been used to analyze the HRN.

This study describes an analytical method for the hypoxia network involving the analysis of the quantitative network indicators of the HIF-1 signaling pathway from the perspective of complex networks. It aims to explore the structural properties of this network and verify the feasibility of the network analytical method. We analyzed the structural attributes of this network after constructing it from the corresponding biomedical database and then checked the complex properties of the network.

## Methods

### Datasets

This study focused on the structure of the hypoxia-inducible factor-1 (HIF-1) (Semenza, 1999; Pugh & Ratcliffe, 2003) signaling pathway of the HRN and retrieved related information from the entries in the Kyoto Encyclopedia of Genes and Genomes (KEGG) database (Kanehisa & Goto, 2000). KEGG provides data resources related to the high-level functions and operations of cells, organs, ecosystems, and other life systems based on molecular-level information, especially information used for parsing molecular datasets generated by gene sequencing and other high-throughput experimental techniques (Kanehisa et al., 2017). There are two essential elements of KEGG discussed in this paper: the KEGG PATHWAY and KEGG ORTHOLOGY databases. The KEGG PATHWAY database is a collection of knowledge-based hand-drawn maps of pathways based on molecular interaction networks covering metabolic, genetic information processing, environmental information processing, cellular processes, biological systems, human diseases, and drug discovery. KEGG PATHWAY provides a concrete explanation of the meaning of the markers of the HIF-1 signaling pathway. The KEGG ORTHOLOGY (KO) database is a set of ortholog assemblages manually defined to denote the nodes (boxes) in the KEGG PATHWAY maps. The distinctive identifier that determines each KO entry is referred to as the K number (‘K’ followed by a five-digit number).

We analyzed the data in the KEGG Markup Language (KGML) file. These data contain information that corresponds to the HIF-1 signaling pathway in KEGG PATHWAY. The KGML, which enables the automated mapping of KEGG pathways, is an extensible markup language (XML) representation of the KEGG pathway. It is conducive to the computer-aided analysis and model building of gene/protein and chemical networks. There are two types of graphic elements in the KGML-based metabolic pathways. One is a rectangle, which represents an enzyme, connected by a “relationship.” The other is a circle, which represents a compound, connected by a “reaction.” In non-metabolic pathways, there are only rectangular elements, which indicates that there are only proteins joined by “relationships” in these pathways. The HIF-1 signaling pathway addressed in this study belongs to the metabolic pathways.

### Overall analytical methods

The methods of this study are based on network analysis. The network of the HIF-1 signaling pathway is a metabolic network. It is a directed unprivileged network; i.e., the direction of a line between two nodes needs to be considered rather than the weight in the network. The corresponding analytical methods applied in this study are as follows: Process the text in the KGML file of the HIF-1 signaling pathway. Refine the network topology information and transform it into a network file that is identifiable for network analysis. Analyze the network indicators and draw the network topology maps. Analyze and test the structural complexity of the network. The generation of the network file is based on regular expression using the text-editing software Notepad++ and functions using the spreadsheet software Excel. The process of network analysis is based on network analysis models using Pajek. These methods and tools are comprehensively utilized to realize the qualitative and quantitative integration analysis of the network structural properties of the HIF-1 signaling pathway.

Biological network analysis tools are used to identify, analyze, visualize, or simulate nodes (reactants) and edges (reactions) from various input data types, including mathematical models of biological networks. There are four commonly used tools, namely Gephi, Networkx, IGraph, and Pajek. All of them are free for use, and they can handle large graph size. Gephi and Pajek are GUI based network analysis tools, whereas Networkx and IGraph are used in a programming language. We have chosen Pajek as the analysis tool based on the following reasons: First, Pajek supports a multi-relational network graph. There are different reactants in the HIF-1 signaling pathway, such as orthologous enzymes, compounds, and groups. And the kind of their relationships is diverse, including activation reactions, expression reactions, and inhibition reactions. Therefore, the network of the HIF-1 signaling pathway is a multi-relational network which can be analyzed by Pajek. Second, Pajek support partition feature enables the identification and extraction of the HIF-1 signaling pathway core network. Third, Pajek supports more network graph layout algorithms, and its algorithms for analyzing the corresponding network features are better. Forth, Pajek is a standalone software that is easy to learn and easy to use (Akhtar, 2014).

### Topology

The network topology indicators include the node number, the line number, the line value, the network density, and the network diameter. The node is an entity that forms the network. The lines between the nodes represent the connections between network entities. The line value is the weight of a line, which indicates the strength of the relationship between network entities. The diameter of the network is the longest path of the shortest distance between pairs of nodes in the network, which is the number of maximum steps required to connect any pair of nodes in the network. The network density is equal to the numeric ratio of the actual connections to the possible connections. It indicates the degree of closeness of the relationships between the nodes in the network.

### Centrality

Centrality is an essential concept of network analysis. A highly centralized network supports the convenient transmission of information. The central node has a critical influence on the transmission of information in the network. The metrics for centrality used in this paper include the centrality of the degree, the closeness, and the betweenness.

#### Degree centrality

The degree centrality of a node is defined as the number of connections of a node. It provides the most intuitive conceptual form of centrality indicator. It can be classified as the overall, input, or output degree centrality. These values correspond to the total number of connections, the number of input connections, and the number of output connections of the node, respectively. The regular formula of the degree centrality is as follows (Wasserman & Faust, 1994):

(1) \begin{eqnarray*}D{C}_{i}=Di{n}_{i}+Dou{t}_{i}\end{eqnarray*}

where *DC*_*i*_ represents the overall degree, *Din*_*i*_ represents the input degree, and *Dout*_*i*_ represents the output degree.

#### Closeness centrality

A node’s closeness centrality is the value obtained by dividing the number of all other nodes by the sum of the geodesic distance between the node and all other nodes. The geodesic distance is the number of connections included in the shortest path between the two nodes. The farther away a node is from other nodes, the lower the closeness centrality of the node is, and vice versa. Similar to the degree centrality, the closeness centrality of the nodes involves the overall closeness centrality, the input closeness centrality, and the output closeness centrality. The closeness centrality of a node reflects the nearness of a node to other nodes. The closer a node is to other nodes, the easier it is for information to reach the node, and the higher its closeness centrality is. The regular formula of the closeness centrality is as follows (Wasserman & Faust, 1994):

(2) \begin{eqnarray*}C{C}_{i}= \frac{N-1}{\sum _{j=1}^{N}{d}_{ij}} \end{eqnarray*}

where *i* ≠ *j*, *N* is the number of nodes, and *d*_*ij*_ is the shortest pathway between nodes *i* and *j*.

#### Betweenness centrality

A node’s betweenness centrality is the ratio of the shortest pathways passing through this node to all of the shortest pathways between any two nodes in the network. The degree centrality and closeness centrality are based on the reachability of a node in the network. In view of betweenness centrality, if this indicator of a node is high, its importance as an intermediary node in the network is higher, and its bridging ability is active.

The normal formula for the betweenness centrality is as follows (Wasserman & Faust, 1994):

(3) \begin{eqnarray*}B{C}_{i}= \frac{2\times \sum _{j\leq k}{g}_{jk}(i)/{g}_{jk}}{(N-1)(N-2)} \end{eqnarray*}

where *i* ≠ *j* ≠ *k*, *g*_*jk*_ represents the number of the shortest pathways between nodes *j* and *k*; *g*_*jk*_(*i*) represents the number of the shortest pathways containing *i*, and *N* represents the number of nodes.

### Cohesive subgroups

Some entities in the network relate to each other so tightly that they form a small local group known as a cohesive subgroup. The number of cohesive subgroups in the network as well as the local and global associations between subgroups are analyzed via cohesive subgroup analysis. This analysis includes component analysis, K-core analysis, and island analysis based on different analytical indicators and perspectives (De Nooy, Mrvar & Batagelj, 2018). The component analysis is mainly applied in this study. The component is an essential indicator of the cohesive subgroup analysis, which refers to the largest connected subnetwork in the network; i.e., there is a way to reach other nodes between any nodes in the subnetwork. Biometabolism networks often contain components that are not connected, and the most significant component is often the one that needs attention.

### Bow-tie structure

The bow-tie property is an essential component of these properties. Broder et al. (2000) revealed that there is a bow-tie structure in the topology structure of the Internet at the macro level.

**Figure 1:**
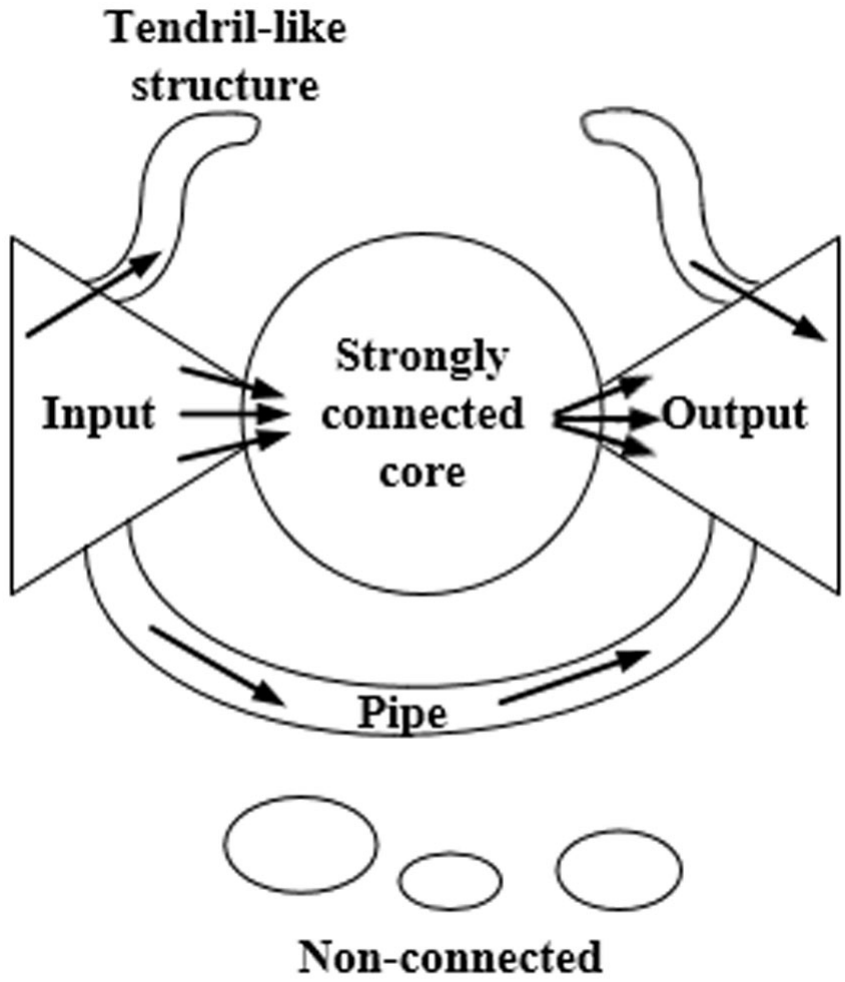
The bow-tie structure of the network. This structure is composed of the input section, the core section of strong connection, the output section, the tendril section, the pipeline section, and the component with no connection.

Figure 1 shows the bow-tie structure. The nodes of the input section can reach the nodes of the core section of a strong connection, while the reverse is not true. The nodes of the core section of the strong connection can reach each other nodes. The nodes of the core section of the strong connection can reach the nodes of the output section, while the reverse is not true. The nodes of the pipe section can reach the nodes of the output section via the input section. The tendril section connects with the input section and the output section. The nodes of the input section can reach the nodes in the same section or the nodes of the output section. There are no connections between the nodes of the non-connected component and other sections.

Ma & Zeng (2003) divided the macrostructure of the biological metabolic network into four parts: the giant strong component (GSC), substrate subset (S), product subset (P), and isolated subset (IS). These components correspond to the strongly connected core, input, output, and disconnected components of the Internet bow-tie structure, respectively, while the exact opposite situation is not observed. Any two metabolites within GSC can be generated from each other by a series of reactions. Any metabolite in S can be converted into the corresponding metabolite in GSC, but not vice versa. Any metabolite in P can be transformed via a series of the corresponding metabolites in GSC, while the exact opposite situation is not observed. The metabolites in IS cannot be converted from those in GSC, nor can they be converted into the corresponding metabolites in GSC.

### Structural complexity

Qian et al. provided a strict definition of complex networks (Xuesen, Jingyuan & Ruwei, 1993). A network with some or all of the following characteristics is known as a complex network. These characteristics are self-organization, self-similarity, attractor, small world, and scale-free. We start with a scale-free perspective to verify the sophisticated attributes of the network. The scale-free property refers to the invariance of the network’s scale, meaning that the degree of the network nodes obeys the law of a power exponential distribution (i.e., the power-law distribution). It is also known as the Pareto distribution rate or the Zipf rate. This distribution law describes the distribution characteristics of the degree of network nodes. A small number of nodes in the network tend to present a large number of connections, but the number of connections of most nodes is very limited. This paper uses the Pareto distribution rate:

(4) \begin{eqnarray*}{P}_{r}[X\geq x]\sim {x}^{-a}\end{eqnarray*}

where *x* is the probability corresponding to the actual measurement scale of the network, and *a* is the scale parameter of the power rate distribution.

The law of the degree distribution of the network nodes needs to be fitted to the power exponential distribution law to verify this characteristic of the core network of the HIF-1 signaling pathway. That is, the fitting value, $\hat {a}$, of the scale parameter, *a*, is obtained. Then, the characteristic is judged by the fitting effect.

### The power-law fitting of the degree distribution

The general method of power-law fitting is to use the least-squares linear fitting based on double-logarithmic coordinates and to use the *R*^2^ test to measure the fitting effect. However, Goldstein et al. believe that the scale result obtained by this method shows a significant error in relation to the corresponding value of the actual measurement network scale (Goldstein, Morris & Yen, 2004). Therefore, Clauset and Barabási et al. proposed a maximum likelihood estimation method for power rate fitting and tested the fitting results using KS statistics (Barabási, Albert & Jeong, 1999; Clauset, Shalizi & Newman, 2009). At present, no research shows that all the data corresponding to the actual measurement scale of the studied network obey the power-law distribution, but there is a critical value. The data corresponding to these scales obey the power-law distribution only when the value corresponding to the actual measurement scale, *x*, is higher than *x*_min_. This study used Clause’s universal method, applicable to both discrete and continuous data, to estimate *x*_min_. For actual network-scale data, the following formula is used to estimate the scale parameter, $\widehat{a}$, of the power-law distribution:

(5) \begin{eqnarray*}\widehat{a}\simeq 1+n{ \left[ \sum _{i=1}^{n}\ln \nolimits \frac{{x}_{i}}{{x}_{\min \nolimits }-0.5} \right] }^{-1}\end{eqnarray*}

where *x*_min_ can be accurately obtained by calculating the maximum difference, *L*, between the actual measurement-scale data and the corresponding data of the fitted model:

(6) \begin{eqnarray*}L=\max _{x\geq \min } \left\vert S(x)-P(x) \right\vert \end{eqnarray*}

where *S*(*x*) is the corresponding data of the actual measurement, *P*(*x*) is the corresponding data of the fitted power-law distribution model, and *x*_min_, which minimizes *L*, is the optimal value.

### Similarity test

This study used the K-S (Kolmogorov-Smirnov) method to test the “distance” *L* between the actual measurement data and the fitted power-law distribution model. The model constructed from actual measurement data is denoted as *M*. This model produces *n* sets of data. There is a set of data whose “distance”, *L*, from the corresponding fitted model, *M*, is greater than the “distance”, *L*, between the actual measured data and the fitted model, *M*. The number of such datasets is *m*. $ \frac{m}{n} $ is represented as *p* and is known as the *p* value. If the *p* value is large (close to 1), it can be considered that the statistical fluctuations alone cause the difference between the actual measurement data and the fitted model. If the *p* value is small, there is room for adjustment in the rationality of the fitted model. If *p* ≤ 0.1, it can be considered that the actual measured data do not obey the power-law distribution (Ping, 2014).

## Results

### Topology analysis

**Figure 2:**
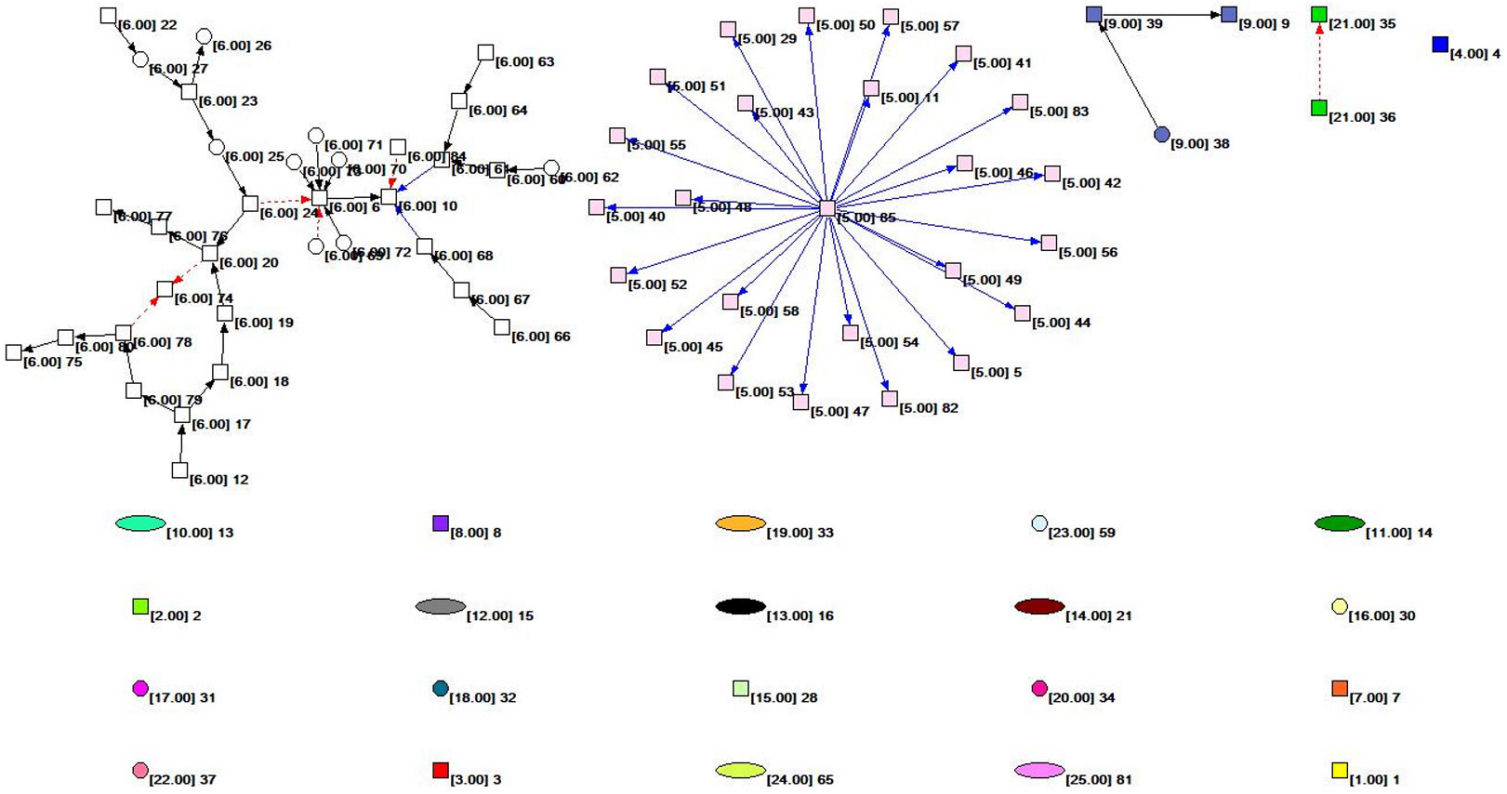
The subnet distribution map of the overall network of the HIF-1 signaling pathway. The numbers beside a node identifies the node. The color of the node and the number in square brackets identify the subnet to which the node belongs. The square nodes, round nodes, and elliptical nodes represent the orthologous enzymes, compounds, and pathways involved in metabolism, respectively. An arrow represents a metabolic association relationship. The shaft of the arrow connects the metabolic reactant, and the arrowhead connects the metabolites. The solid black line indicates the “activated” metabolic behavior, while the solid blue line indicates the “expression” metabolic behavior. Moreover, the red dashed line indicates “suppressive” metabolic behavior.

Figure 2 shows the overall topology of the HIF-1 signaling pathway network based on the KGML file from KEGG. Quantitative analysis of the network indicators is performed, and the results are as follows: The numbers of nodes and lines are 85 and 61, respectively. The network has a ring and ringless density of 0.00844291 and 0.00854342, respectively. The average node degree (i.e., the average number of connections) is 1.43529412. The network diameter is 7, and the average distance between the nodes is 2.26761. There are 59 orthologous enzymes, 16 compounds, eight pathways, and two unknown groups in this metabolic network that have no references in KEGG. The numbers of activation reactions, expression reactions, and inhibition reactions are 29, 26, and 6, respectively.

**Figure 3:**
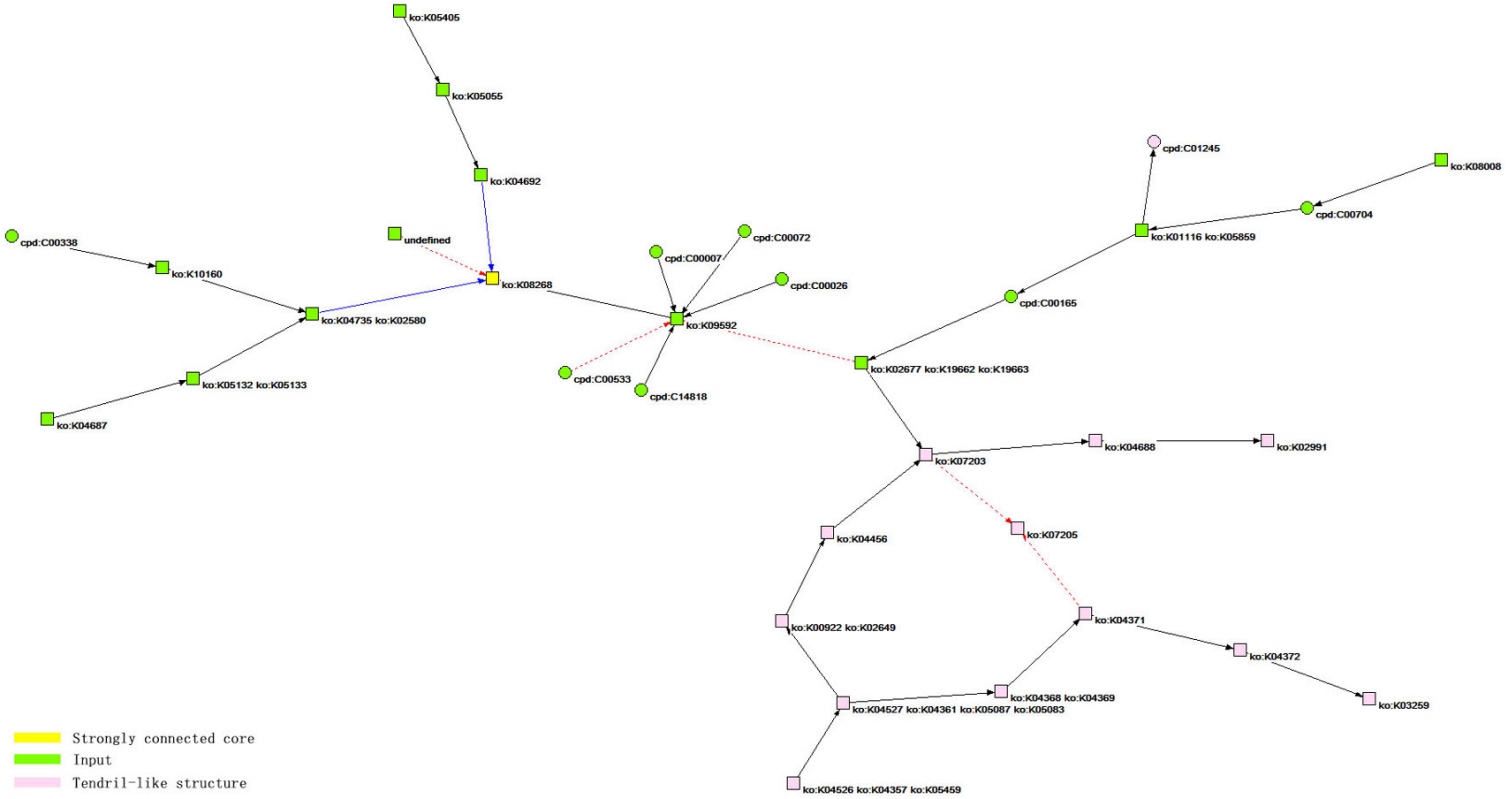
The core network bow-tie structure of the HIF-1 signaling pathway. According to the analysis results, the bow-tie structure of the core network of the HIF-1 signaling pathway includes three types of components. The first is a strongly connected core, corresponding to the yellow node, ko: K08268, in the figure, and the corresponding reactant in the KEGG database is HIF-1 *α*. The second is the input components, including 20 green nodes, such as ko: K09592, corresponding to 20 reactants, such as PHD (EGLN, HPH), in the KEGG database. The third is a tendril-like structure including 13 pink nodes, such as ko: K04526, ko: K04357, and ko: K05459, which correspond to 13 reactants, such as GF (INS, EGF, IGF1), in the KEGG database.

The core network of the HIF-1 signaling pathway is identified based on cohesive subgroup analysis. The network is extracted for the convenience of further study, as shown in Fig. 3. The quantitative indicators of the network are as follows: the number of nodes is 34, and the number of lines is 34. The network has a ring density of 0.02941176 and a ringless density of 0.03030303. The average node degree is 2. The network diameter is 7, and the average distance between the nodes is 2.57018. There are 24 orthologous enzymes and 10 compounds in this metabolic network. The number of unknown groups that have no references in KEGG is 1; the number of activation reactions is 27; the number of expression reactions is 2; and the number of inhibition reactions is 5.

### Centrality analysis

#### Degree centrality

The degree centrality of the overall network of the HIF-1 signaling pathway is analyzed and includes the overall centrality, the input centrality, and the output centrality. The top 10 nodes are listed in Table 1.

Four nodes appeared in the top 10 list in terms of the three-degree centrality indicators, numbered 20, 60, 61 and 78. They were serine/threonine-protein kinase mTOR(node 20), toll-like receptor 4(node 60), transcription factor p65, nuclear factor NF-kappa-B p105 subunit(node 61), and mitogen-activated protein kinase 1/3(node 78), respectively.

#### Closeness centrality

The analysis of the closeness centrality of the overall network of the HIF-1 signaling pathway is performed to calculate the total closeness centrality, the input closeness centrality, and the output closeness centrality. Table 2 lists the top 10 nodes in terms of the closeness centrality. There are two nodes with both the overall and the input closeness centrality ranked in the top 10, numbered 29 and 82. They are pyruvate dehydrogenase kinase isoform 1 and apoptosis regulator Bcl-2, respectively. The number of the node with the overall and output closeness centrality in the top 10 is 85, which is an undefined complex of gene products in KEGG. The node with the input closeness centrality and output closeness centrality ranked in the top 10 is numbered 20, which is serine/threonine-protein kinase mTOR.

**Table 1:** Top 10 nodes of the overall network degree centrality of the HIF-1 signaling pathway.

Rank	Overall degree centrality		Input degree centrality		Output degree centrality	
	Node number	Value	Node number	Value	Node number	Value
1	85	24	6	6	85	24
2	6	7	10	4	24	2
3	10	4	61	2	23	2
4	20	4	20	2	20	2
5	61	3	74	2	78	2
6	24	3	60	1	17	2
7	23	3	80	1	63	1
8	78	3	29	1	62	1
9	17	3	78	1	61	1
10	60	2	58	1	60	1

**Table 2:** Top 10 nodes of the overall network closeness degree centrality of the HIF-1 signaling pathway.

Rank	Overall closeness centrality		Input closeness centrality		Output closeness centrality	
	Node number	Value	Node number	Value	Node number	Value
1	85	0.2941	10	0.1008	85	0.2941
2	29	0.1502	6	0.0647	17	0.0462
3	58	0.1502	74	0.051	24	0.0449
4	57	0.1502	20	0.0424	12	0.0398
5	82	0.1502	61	0.0392	23	0.0392
6	56	0.1502	76	0.037	25	0.0366
7	55	0.1502	77	0.0338	20	0.0353
8	54	0.1502	82	0.0235	78	0.0353
9	53	0.1502	60	0.0235	27	0.035
10	52	0.1502	29	0.0235	22	0.0324

#### Betweenness centrality

Table 3 shows the betweenness analysis results of the overall network of the HIF-1 signaling pathway.

**Table 3:** Top 10 nodes of the overall network betweenness degree centrality of the HIF-1 signaling pathway.

Rank	Node number	Value
1	20	0.0036
2	24	0.0034
3	25	0.003
4	23	0.0026
5	27	0.0014
6	6	0.0014
7	19	0.0014
8	76	0.0014
9	17	0.0014
10	78	0.0013

Two nodes appeared in the top 10 list in terms of the betweenness centrality and degree centrality, numbered 20 and 78, which are serine/threonine-protein kinase mTOR and mitogen-activated protein kinase 1/3, respectively.

### Cohesive subgroup analysis

First, the distribution of the subset of the overall network is determined. Then, the core network based on the distribution is determined. The minimum component size is 1; i.e., the smallest subnet can be an isolated node without connections. Table 4 shows the analysis results.

**Table 4:** The maximum connected subnet distribution of the overall network of the HIF-1 signaling pathway.

Subnet number	Number of nodes included		Cumulative number of nodes		Representative network node number
	*n*	%	*n*	%	
1	1	1.1765	1	1.1765	1
2	1	1.1765	2	2.3529	2
3	1	1.1765	3	3.5294	3
4	1	1.1765	4	4.7059	4
5	25	29.4118	29	34.1176	5
6	34	40	63	74.1176	6
7	1	1.1765	64	75.2941	7
8	1	1.1765	65	76.4706	8
9	3	3.5294	68	80	9
10	1	1.1765	69	81.1765	13
11	1	1.1765	70	82.3529	14
12	1	1.1765	71	83.5294	15
13	1	1.1765	72	84.7059	16
14	1	1.1765	73	85.8824	21
15	1	1.1765	74	87.0588	11
16	1	1.1765	75	88.2353	30
17	1	1.1765	76	89.4118	31
18	1	1.1765	77	90.5882	32
19	1	1.1765	78	91.7647	33
20	1	1.1765	79	92.9412	34
21	2	2.3529	81	95.2941	35
22	1	1.1765	82	96.4706	37
23	1	1.1765	83	97.6471	59
24	1	1.1765	84	98.8235	65
25	1	1.1765	85	100	81

The data in the table show that the overall network of the HIF-1 signaling pathway includes 25 subnets. The minimum size of the subnet is 1; i.e., there is only one node in the subnet. The maximum size is 34; i.e., 34 nodes are in the subnet.

We colored different subnets differently for the convenience of analysis in this paper. The number of the subnet to which the node belongs is identified. The subnet distribution map of the overall network of the HIF-1 signaling pathway is obtained based on Pajek, as shown in Fig. 2. Figure 2 depicts the subnet distribution of the overall network of the HIF-1 signaling pathway graphically. For example, “[6.00] 20” means that node 20 belongs to the number 6 subnet.

Table 4 and Fig. 2 show that the number 6 subnet contains most nodes of all the subnets and has meaningful reaction relationships. Therefore, subnet 6 is the core network of the HIF-1 signaling pathway.

### Bow-tie structure analysis

The Bow-tie analysis module of Pajek software was used to analyze the macrostructure of the core network of the HIF-1 signaling pathway. Table 5 shows the distribution of the structure.

We colored the different structures in the core network topology of the HIF-1 signaling pathway, as shown in Fig. 3.

The specification of the vertices of the core component of the HIF-1 signaling pathway is shown in Table 6.

**Table 5:** The quantity distribution of the nodes in the core network of the HIF-1 signaling pathway.

Structure category	Frequency of occurrence		Cumulative frequency		Representative reactants
	*n*	%	*n*	%	
The core section of strong connection	1	2.9412	1	2.9412	hypoxia-inducible factor 1 alpha
The input section	20	58.8235	21	61.7647	hypoxia-inducible factor prolyl hydroxylase
The tendril-like section	13	38.2353	34	100	Insulin, epidermal growth factor, insulin-like growth factor 1

**Table 6:** The biological meanings of the vertices in the core network of the HIF-1 signaling pathway.

**Vertices**	**Entry**	**Name**	**Definition(ko) or Comment(cpd)**	**Classificationh of Bow-tie structure**
1	ko:K09592	EGLN, HPH	hypoxia-inducible factor prolyl hydroxylase [EC:1.14.11.29]	IN
2	ko:K08268	HIF1A	hypoxia-inducible factor 1 alpha	LSSC: Largest Strongly Connected Component
3	ko:K04526	INS	insulin	TENDRILS
3	ko:K04357	EGF	epidermal growth factor	TENDRILS
3	ko:K05459	IGF1	insulin-like growth factor 1	TENDRILS
4	ko:K04527	INSR, CD220	insulin receptor [EC:2.7.10.1]	TENDRILS
4	ko:K04361	EGFR, ERBB1	epidermal growth factor receptor [EC:2.7.10.1]	TENDRILS
4	ko:K05087	IGF1R, CD221	insulin-like growth factor 1 receptor [EC:2.7.10.1]	TENDRILS
4	ko:K05083	ERBB2, HER2, CD340	receptor tyrosine-protein kinase erbB-2 [EC:2.7.10.1]	TENDRILS
5	ko:K02649	PIK3R1_2_3	phosphoinositide-3-kinase regulatory subunit alpha/beta/delta	TENDRILS
5	ko:K00922	PIK3CA_B_D	phosphatidylinositol-4,5-bisphosphate 3-kinase catalytic subunit alpha/beta/delta [EC:2.7.1.153]	TENDRILS
6	ko:K04456	AKT	RAC serine/threonine-protein kinase [EC:2.7.11.1]	TENDRILS
7	ko:K07203	MTOR, FRAP, TOR	serine/threonine-protein kinase mTOR [EC:2.7.11.1]	TENDRILS
8	ko:K08008	NOX1, MOX1	NADPH oxidase 1	IN
9	ko:K01116	PLCG1	phosphatidylinositol phospholipase C, gamma-1 [EC:3.1.4.11]	IN
9	ko:K05859	PLCG2	phosphatidylinositol phospholipase C, gamma-2 [EC:3.1.4.11]	IN
10	ko:K02677	PRKCA	classical protein kinase C alpha type [EC:2.7.11.13]	IN
10	ko:K19662	PRKCB	classical protein kinase C beta type [EC:2.7.11.13]	IN
10	ko:K19663	PRKCG	classical protein kinase C gamma type [EC:2.7.11.13]	IN
11	cpd:C00165	Diacylglycerol;Diglyceride	Generic compound in reaction hierarchyIncluding 1,2-Diacyl-sn-glycerol [CPD:C00641] and 2,3-Diacyl-sn-glycerol	IN
12	cpd:C01245	D-myo-Inositol 1,4,5-trisphosphate;1D-myo-Inositol 1,4,5-trisphosphate;Inositol 1,4,5-trisphosphate;Ins(1,4,5)P3		TENDRILS
13	cpd:C00704	Superoxide;O2.-;Superoxide anion;O2-		IN
14	ko:K10160	TLR4, CD284	toll-like receptor 4	IN
15	ko:K04735	RELA	transcription factor p65	IN
15	ko:K02580	NFKB1	nuclear factor NF-kappa-B p105 subunit	IN
16	cpd:C00338	Lipopolysaccharide;LPS		IN
17	ko:K04687	IFNG	interferon gamma	IN
18	ko:K05132	IFNGR1, CD119	interferon gamma receptor 1	IN
18	ko:K05133	IFNGR2	interferon gamma receptor 2	IN
19	ko:K05405	IL6	interleukin 6	IN
20	ko:K05055	IL6R, CD126	interleukin 6 receptor	IN
21	ko:K04692	STAT3	signal transducer and activator of transcription 3	IN
22	cpd:C00533	Nitric oxide;NO;Nitrogen monoxide		IN
23	cpd:C00007	Oxygen;O2		IN
24	cpd:C14818	Fe2+;Fe(II);Ferrous ion;Iron(2+)		IN
25	cpd:C00072	Ascorbate;Ascorbic acid;L-Ascorbate;L-Ascorbic acid;Vitamin C		IN
26	cpd:C00026	2-Oxoglutarate;Oxoglutaric acid;2-Ketoglutaric acid;alpha-Ketoglutaric acid		IN
27	ko:K07205	EIF4EBP1	eukaryotic translation initiation factor 4E binding protein 1	TENDRILS
28	ko:K03259	EIF4E	translation initiation factor 4E	TENDRILS
29	ko:K04688	RPS6KB	ribosomal protein S6 kinase beta [EC:2.7.11.1]	TENDRILS
30	ko:K02991	RP-S6e, RPS6	small subunit ribosomal protein S6e	TENDRILS
31	ko:K04371	ERK, MAPK1_3	mitogen-activated protein kinase 1/3 [EC:2.7.11.24]	TENDRILS
32	ko:K04368	MAP2K1, MEK1	mitogen-activated protein kinase kinase 1 [EC:2.7.12.2]	TENDRILS
32	ko:K04369	MAP2K2, MEK2	mitogen-activated protein kinase kinase 2 [EC:2.7.12.2]	TENDRILS
33	ko:K04372	MKNK, MNK	MAP kinase interacting serine/threonine kinase [EC:2.7.11.1]	TENDRILS
34	undefined			IN

### Structural complexity analysis

We analyzed the distribution of network node degrees. That is, the scale of the core network of the HIF-1 signaling pathway is measured. The degree distribution of the core network of the HIF-1 signaling pathway is shown in Table 7.

**Table 7:** The degree distribution of the core network of the HIF-1 signaling pathway.

Degree	Frequency of occurrence		Cumulative frequency		Representative reactants
	*n*	%	*n*	%	
1	14	41.1765	14	41.1765	Insulin, epidermal growth factor, insulin-like growth factor 1
2	12	35.2941	26	76.4706	phosphatidylinositol-4,5-bisphosphate 3-kinase catalytic subunit alpha/beta/delta, phosphoinositide-3-kinase regulatory subunit alpha/beta/delta
3	5	14.7059	31	91.1765	insulin receptor, epidermal growth factor receptor, insulin-like growth factor 1 receptor, receptor tyrosine-protein kinase erbB-2
4	2	5.8824	33	97.0588	hypoxia-inducible factor 1 alpha
7	1	2.9412	34	100	hypoxia-inducible factor prolyl hydroxylase

**Figure 4:**
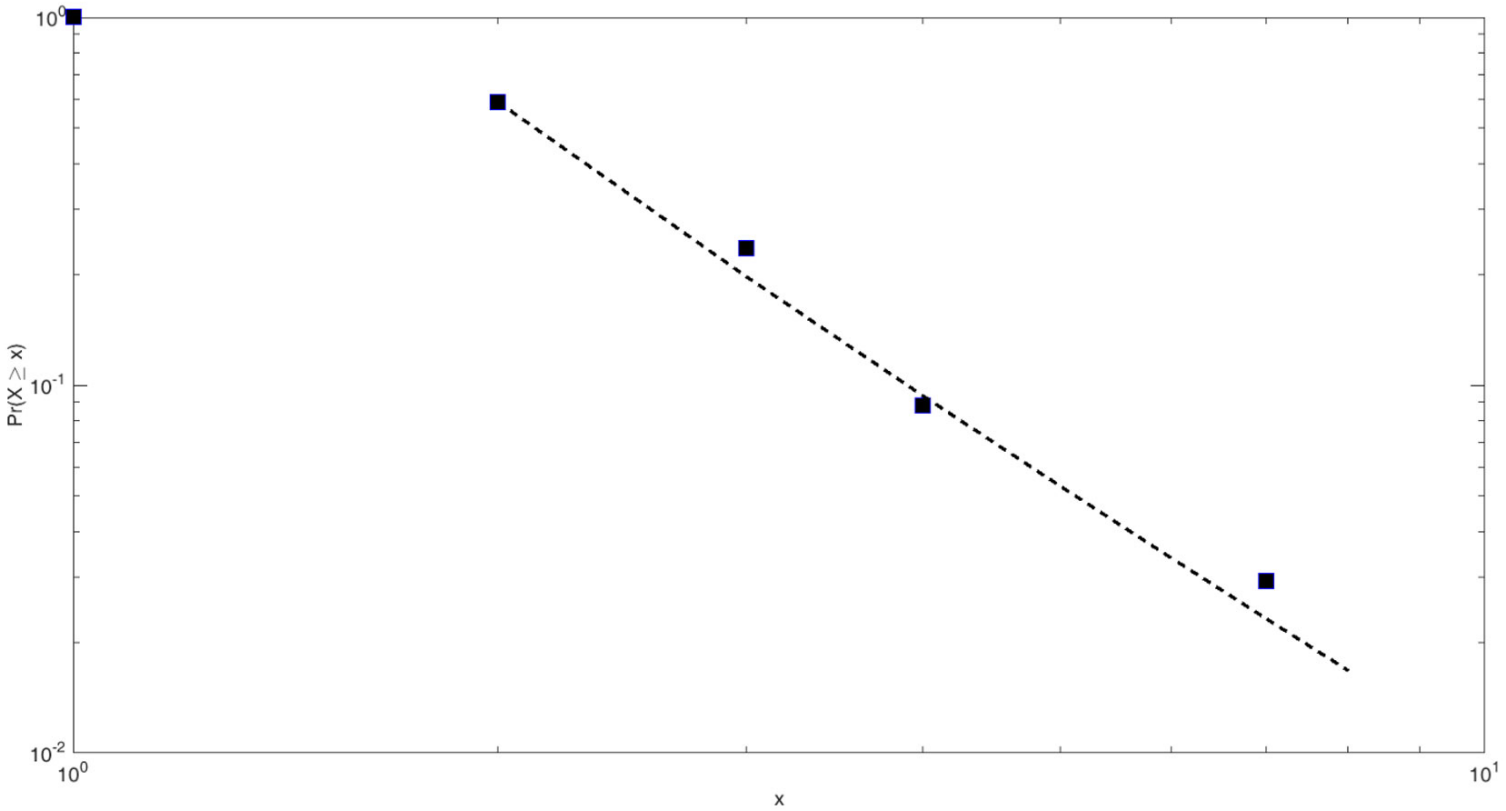
Power-law fitting of the core network scale of the HIF-1 signaling pathway. The abscissa corresponds to the network scale. The ordinate is the distribution rate of the network scale. The black square points are the actual measured power-law distribution points. The dotted line is the slope of the fitted straight line.

Based on the above methods, the power-law fitting results of the core network scale of the HIF-1 signaling pathway in this study are shown in Fig. 4.

Based on Clauset’s method, the power-law fitting model for the core network of the HIF-1 signaling pathway can be calculated as follows:

(7) \begin{eqnarray*}{P}_{r}[X\geq x]\sim {x}^{-3.28}\end{eqnarray*}

The scale parameter of the power-law distribution of the core network of the HIF-1 signaling pathway is 3.28, and *x*_min_ = 2. That is, the nodes with a degree of 2 or more in the network obey the Pareto distribution rate. The similarity detection index of this fitted model is much larger than 0.1 and closer to 1. This indicates that the fitting effect is good.

## Discussion

The overall network centrality analysis of the HIF-1 signaling pathway suggests that K07203 appears in the top 10 list in terms of the betweenness centrality, input closeness centrality, and output closeness centrality. It corresponds to the mTOR enzyme, which is known as an essential element in the field of HRN research. These results suggest that the mTOR enzyme has a crucial influence on HIF-1 signaling, which conforms to the known findings, indicating the effectiveness of the network analytical method.

With regard to the component analysis of the overall network of the HIF-1 signaling pathway, the largest connected subnet of the overall network of the HIF-1 signaling pathway is the number 6 subnet; i.e., the number 6 subnet is the core network of the HIF-1 signaling pathway, which is consistent with the findings of current biomedical research.

With respect to the bow-tie structure analysis, the work scope of Ma et al. does not involve the macrostructure analysis of the core network of the HIF-1 signaling pathway (Ma & Zeng, 2003). Therefore, this study can be regarded as a supplement to their work and as a verification of their conclusions. The core network of the HIF-1 signaling pathway has a bow-tie structure. It includes macrostructures such as a strongly connected core, an input, and a tendril-like structure. It is worth noting that there are no output components in the structure; that is, HIF-1α has no metabolites. The reason is that the corresponding orthologous enzymes are not listed in the KGML file of KEGG. In KEGG’s hand-painted HIF-1 signaling pathway diagram, HIF-1α has two output pathways: aerobic degradation and hypoxic DNA expression. However, there are no orthologous enzyme products. Therefore, the diagram is consistent with the analysis results obtained by this study. According to the analysis, HIF-1α is the strongly connected core of the bow-tie structure in the core network of the signaling pathway. The bow-tie structure of the core network of the HIF-1 signaling pathway has no formal output components. However, the actual judgment based on a priori knowledge of the degradation pathway of HIF-1α under normoxic conditions suggests that the bow-tie structure of the network contains a potential output structure. Moreover, this structure is HIF-1α itself. In this sense, the bow-tie structure of the core network of the HIF-1 signaling pathway is relatively complete. Additionally, this study showed that the core network of the HIF-1 signaling pathway has a tendril-like structure. This finding further verifies the similarity of the biological metabolic network and the Internet in terms of macroscopic structure, based on the work of Ma et al.

The results of the structural complexity analysis show that the core network of the HIF-1 signaling pathway has scale-free characteristics. Therefore, the HIF-1 signaling pathway network is a relatively typical complex network. It can be studied using complexity-based research methods.

We plan to analyze the HIF-1 signaling pathway network from a network perspective since the network analytical method is systematic and holistic. It is efficient in analyzing complex biological networks, and the HIF-1 signaling pathway is an essential component of the HRNs. However, the present study has a few limitations. For example, to verify the network analytical method’s effectiveness, we only analyzed the structural information of the network of the HIF-1 signaling pathway, which is insufficient to explore the unknown critical elements and the relationships of the elements of the pathway. Therefore, further studies need to be carried out to investigate the complex interactions of the elements of the HRNs at different levels using a network analytical method so that new relationships and critical elements of the networks can be identified. Meanwhile, as hypoxia is a dynamic process, the hypoxia response network’s evolution involves the time element as an essential feature. Our work focused on the hypoxia network’s topological features at a certain point in time. We can analyze the network’s evolution from the following aspects: The first is the statistical analysis of the network’s structural data. We can treat these data at a specific time as a snapshot. Then snapshots of the same network from different time intervals can be taken, observed, and analyzed as time evolves. The second is utilizing simulation to address network dynamics issues. We can use Petri net-based modeling and other forms of simulations to explore how networks evolve and adapt and the impact of interventions on those networks (Li et al., 2016). Finally, we analyzed the HIF-1 signaling pathway using information retrieved from the Kyoto Encyclopedia of Genes and Genomes (KEGG) based on this study’s network analytical method. A similar analysis can also be performed to analyze other biological networks such as the recent genome-wide regulatory network for hypoxia adaption to identify the network elements’ interaction from a holistic view and get some interesting findings (Xin et al., 2020).

## Conclusions

In this study, we investigated the HRN from the perspective of complex networks. In the analysis of the overall network, we identified the topological properties of the overall network of the HIF-1 signaling pathway and the biomedical meaning of the network indicators based on the construction of the network using the data retrieved from the KEGG database. The centrality indicators of the overall network, the remarkable nodes, and the biomedical meaning of these nodes could be identified through centrality analysis. Based on component analysis, the cohesive subgroups of the overall network were obtained; the different subnets were indicated; and the core HRN was identified. In the analysis of the core network, we refined the core network of the HIF-1 signaling pathway via topology analysis of the network information based on the overall network analysis. We obtained a topological structure diagram and the quantitative indicators of this network. Then, we determined the bow-tie structure existing in this network through the analysis of the bow-tie structure. This structure is composed of three types of components: the core structure, the input structure, and the tendril-like structure. We can verify and further add to the existing research conclusions by analyzing each part’s node distribution. Additionally, a power-law fitting model of this network was constructed through the verification of its complex attributes. The results showed that the power-law fitting model of the core network of the HIF-1 signaling pathway presented a good fitting effect. This proved that the core network of the HIF-1 signaling pathway has scale-free characteristics and is a relatively typical complex network. We are aware that our research may have two limitations. The first is some reactants in the network undefined in KEGG, which prevent in-depth recognition of HRN. The second is the network analysis performed in this research confined to the HIF-signaling pathway, while other essential pathways of HRN are not analyzed. These limitations highlight the direction for follow-up in-depth research. Although this network analysis is preliminary work, it enriches and improves upon the existing research conclusions. It provides a basis for in-depth research on HRNs at different levels using network analytical methods. In particular, these results can be applied in parallel with traditional studies of the HRN, which will promote and complement each other.

##  Supplemental Information

10.7717/peerj.10985/supp-1 Supplemental Information 1 Network raw data

## Abbreviations

HRN: Hypoxia response network
HIF-1: Hypoxia-inducible factor-1
KEGG: Kyoto Encyclopedia of Genes and Genomes
KO: KEGG Orthology
KGML: KEGG Markeup Language
XML: Extensible Markeup Language

## Additional Information and Declarations

### Data Availability

The following information was supplied regarding data availability: Data is available at KEGG, hsa04066. The xml file (hsa04066.xml) can be downloaded by clicking ’Download KGML’.
https://www.kegg.jp/kegg-bin/show_pathway?hsa04066.
